# Studentische Ausbildung im Fach Rechtsmedizin in Deutschland: Prüfungen und Evaluation

**DOI:** 10.1007/s00194-021-00454-z

**Published:** 2021-02-15

**Authors:** Sibylle Nold, Steffen Heide, Thomas Bajanowski, Sven Anders

**Affiliations:** 1 Institut für Rechtsmedizin, Universitätsklinikum Hamburg-Eppendorf, Butenfeld 34, 22529 Hamburg, Deutschland; 2 Institut für Rechtsmedizin, Universitätsklinikum Dresden, Dresden, Deutschland; 3 Institut für Rechtsmedizin, Universitätsklinikum Essen, Essen, Deutschland

**Keywords:** Rechtsmedizinische Lehre, Medizindidaktik, Medizinstudium, Teaching in forensic medicine, Didactics of medicine, Medical school

## Abstract

Seit der Umsetzung der Approbationsordnung für Ärzte 2002 haben sich erhebliche Veränderungen in den Bereichen Lehre, Prüfungen und Evaluation ergeben. Zur Erfassung der aktuellen Situation im Fach Rechtsmedizin erfolgte eine standardisierte Befragung unter den rechtsmedizinischen Instituten in Deutschland mittels eines Online-Fragebogens. Der Rücklauf betrug 80 %. Die Ergebnisse der Befragung weisen auf ein Überwiegen faktenbasierter Prüfungen hin. Anpassungen an die häufig praktisch ausgerichteten Unterrichtsformate erscheinen hier erforderlich. Die Evaluationsergebnisse zeigen überwiegend eine hohe studentische Zufriedenheit mit der Lehre im Fach Rechtsmedizin. Famulaturen und praktisches Jahr können aktuell von etwa 90 % der Institute angeboten werden. Die für die Lehre zur Verfügung stehende Stundenzahl wird von einem relevanten Teil der befragten Institute als nicht ausreichend bewertet.

## Abstract (en)

The implementation of the Medical Licensure Act in 2002 led to remarkable changes in teaching, testing and evaluation in undergraduate medical education. Using an online questionnaire the current situation among German institutes for forensic medicine was evaluated. The return rate of the questionnaires was 80%. The results point at a preponderance of testing of factual knowledge. A change to testing of practical skills appears necessary to match the learning objectives of practical teaching. The evaluation results represent a high level of student contentment with teaching in forensic medicine. Clinical electives can be offered by more of 90% of the institutes. Teaching time in forensic medicine is thought to be inadequate by a relevant number of institutes.

## Einleitung

Seit der Novellierung der Approbationsordnung für Ärzte im Jahr 2002 [1] hat an der überwiegenden Zahl der deutschen medizinischen Fakultäten ein curricularer Reformprozess stattgefunden, der sich auch in Änderungen der rechtsmedizinischen Lehre im Rahmen des Medizinstudiums widerspiegelt. Kurze Zeit nach dem Inkrafttreten der Novellierung erfolgte 2006 eine fragebogenbasierte Untersuchung zum damaligen Status quo der Lehre in den deutschen rechtsmedizinischen Instituten [2]. Zwar zeigte sich schon damals z. B. ein deutlicher Trend zu einer Standardisierung des Unterrichtsinhalts an den Instituten, Lernzielkataloge lagen aber nur in 25 % vor.

Da seit der damaligen Erhebung die curricularen Änderungsprozesse fortgeschritten sind, vielerorts unter Einbindung medizindidaktischer Prinzipien, ist von einem gegenüber dem damaligen Stand veränderten Bild auszugehen. Vor dem Hintergrund absehbarer zukünftiger Veränderungen des gesetzlichen Rahmens der Medizinerausbildung handelt es sich hierbei naturgemäß um einen dynamischen, sich weiterentwickelnden Prozess: So liegt seit 2019 ein Entwurf zu einer Novellierung der aktuell gültigen Approbationsordnung vor, der im Rahmen des Masterplans Medizin 2020 zu sehen ist und u. a. auf eine stärker praxisnahe Gestaltung der universitären Ausbildung zukünftiger Mediziner fokussiert [3].

Um eine aktualisierte Darstellung der studentischen Ausbildung im Fach Rechtsmedizin in Deutschland zu erhalten, erfolgte in Anlehnung an die Erhebung aus dem Jahre 2006 eine aktualisierte und erweiterte Befragung der universitären rechtsmedizinischen Institute. Die Ergebnisse der im Jahre 2019 durchgeführten Online-Befragung hinsichtlich der inhaltlichen und strukturellen Aspekte der rechtsmedizinischen Lehrveranstaltungen wurden bereits in einer ersten Publikation dargestellt [4].

Einen zweiten Schwerpunkt der Befragung stellte die gegenwärtige Situation auf den Gebieten rechtsmedizinischer Prüfungen und der Evaluation sowie der aktuellen Möglichkeit zur Durchführung von Famulaturen und praktischem Jahr dar.

## Material und Methoden

Für die Datenerhebung wurde ein Fragebogen mit 64 Items (Einzelfragen) konzipiert, von denen 13 für die vorliegende Arbeit ausgewertet wurden. Die übrigen 51 Items zu inhaltlichen und strukturellen Aspekten der Lehre wurden bereits gesondert betrachtet und publiziert [4]. Der Fragebogen deckte die folgenden Themenbereiche ab:

- Prüfungsmethoden,
- Prüfungsvorbereitung,
- Evaluation der rechtsmedizinischen Unterrichtsveranstaltungen,
- subjektive Beurteilung der rechtsmedizinischen Lehre,
- Famulatur und Praktisches Jahr.

Die Fragen orientierten sich teilweise an der Erhebung von 2006 [2], wurden ggf. modifiziert und erweitert sowie um aktuelle Aspekte ergänzt. Der Fragebogen wurde im Sinne eines Pretests durch vier in der universitären Rechtsmedizin ärztlich tätige Personen auf Verständlichkeit geprüft und aufgrund der Rückmeldungen im Detail angepasst.

Durch eine Internetrecherche, ggf. ergänzt durch eine klärende telefonische Kontaktaufnahme, wurden die Lehrbeauftragten der universitären rechtsmedizinischen Institute in Deutschland ermittelt. Die Datenerhebung erfolgte mittels eines Online-Fragebogens (SoSci Survey GmbH, München, Deutschland; www.soscisurvey.com) im Zeitraum vom 11.06.2019 bis zum 31.06.2019. Den Lehrbeauftragten wurde per Mail ein entsprechender Link zu der Befragung übermittelt. Im Vorwege wurden sowohl die Lehrbeauftragten als auch die Direktorinnen und Direktoren der Institute schriftlich über die Befragung informiert.

Jedes der nachfolgend genannten Institute erhielt einen individuellen Link, Institute mit 2 Standorten (Erlangen, Essen, Gießen, Halle, Heidelberg, Kiel, Köln, München) erhielten 2 getrennte Links für jeden Standort (Tab. 1).

**Table 1:** 

1. Berlin	15. Heidelberg – Mannheim
2. Bonn	16. Homburg/Saar
3. Dresden	17. Jena
4. Düsseldorf	18. Kiel – Lübeck
5. Erlangen – Regensburg	19. Köln – Aachen
6. Essen – Bochum	20. Leipzig
7. Frankfurt/M.	21. Mainz
8. Freiburg	22. München LMU – TU
9. Gießen – Marburg	23. Münster
10. Göttingen	24. Rostock
11. Greifswald	25. Tübingen
12. Halle – Magdeburg	26. Ulm
13. Hamburg	27. Würzburg
14. Hannover	–

Die Auswertung erfolgte deskriptiv mithilfe von MS Office Excel 2013 (Microsoft Corporation, WA, USA).

## Ergebnisse

Zwei der Institute mit 2 Standorten füllten jeweils lediglich einen Fragebogen aus, 4 der in Tab. 1 genannten Institute, davon eines mit 2 Standorten, beteiligten sich nicht an der Umfrage. Somit betrug der Rücklauf 28 von 35 Fragebogen (80 %).

### Prüfungsmethoden

**Figure 1:**
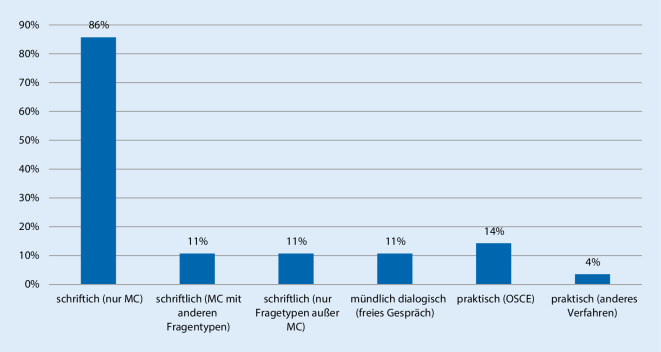

Bei den Prüfungsmethoden der fakultätsinternen Prüfungen im Fach Rechtsmedizin dominiert mit 86 % (*n* = 24) aller Rückmeldungen die Durchführung von alleinigen Multiple-Choice(MC)-Klausuren (Abb. 1). An jeweils 3 Standorten (11 %) werden MC-Fragen mit anderen Fragetypen kombiniert oder alleinig andere Fragetypen in schriftlichen Prüfungen eingesetzt (z. B. „short answer questions“ [SAQ]). Weitere 3 Standorte prüfen mündlich-dialogisch in einem freien Gespräch. Praktische Prüfungen werden an 5 Standorten durchgeführt, in 4 Fällen in Form einer „objective structured clinical examination“ (OSCE), in einem Fall in Form einer Checklisten-basierten strukturierten Einzelprüfung am realen Leichnam. Bei der Beantwortung dieser Frage waren aufgrund der denkbaren Kombination verschiedener Prüfungsformate Mehrfachnennungen möglich.

An 21 Standorten (75 %) findet die Prüfung in einer Großgruppe statt (gesamte Semesterkohorte), jeweils 2 Institute führen Einzel- oder Kleingruppenprüfungen durch. Eine Kombination aus Prüfungen der gesamten Semesterkohorte und Einzel- oder Kleingruppenprüfungen gaben 3 Standorte an.

Bei der Prüfungsauswertung dominiert eine automatisierte Auswertung papierbasierter Prüfungen (*n* = 14, 50 %). Eine händische Auswertung erfolgt in 7 Instituten (25 %). Eine rein computerbasierte E‑Klausur wird von 9 Standorten durchgeführt (32 %). Bei der Beantwortung dieser Frage waren aufgrund der denkbaren Kombination verschiedener Prüfungsformate Mehrfachnennungen möglich.

Ein „Knock-out“-Item, eine Frage, bei deren Falschbeantwortung die gesamte Prüfung als nichtbestanden gilt, findet nur an einer Fakultät Verwendung.

Die Frage nach der Durchführung einer Itemanalyse der schriftlichen fakultätsinternen Prüfungen wurde von 75 % (*n* = 21) der Befragten bejaht und in 3 Fällen verneint; 4 der Befragten konnten dieses Item nicht beantworten, da ihnen unbekannt war, ob eine Itemanalyse erfolgt.

### Evaluation

Eine studentische Lehrevaluation erfolgt an 27 der 28 teilnehmenden Standorte. Überwiegend erfolgt die Evaluation nach dem Semester- bzw. Modulende (*n* = 21, 78 %), an 5 Fakultäten liegt der Evaluationszeitpunkt direkt nach der jeweiligen Lehrveranstaltung, in einem Fall zu Beginn des folgenden Semesters. In etwa zwei Dritteln der Fälle erfolgt die Evaluation rein einrichtungsbezogen (*n* = 18, 67 %), 8 Fakultäten evaluieren sowohl einrichtungs- als auch dozentenbezogen. In nur einem Fall erfolgt eine rein dozentenbezogene Evaluation.

Die Anzahl der Evaluationsitems schwankte dabei erheblich und lag zwischen 3 und 46 Einzelitems. Ebenso stark differiert die Angabe, wie groß der Anteil der Studierenden ist, die sich an der Evaluation an den jeweiligen Fakultäten beteiligen. Dieser liegt zwischen 1,5 und 100 %, mit einem Durchschnittswert von 47,8 %.

Ein fakultätsinternes, fächerbezogenes Evaluationsranking war den Befragten an 16 Standorten bekannt (59 %). Hierbei belegten 12 Institute einen der 10 besten Plätze, in 6 Fällen Platz 1 (teils veranstaltungsabhängig).

### Praktisches Jahr (PJ) und Famulatur

Famulaturen waren zum Zeitpunkt der Datenerhebung an dem Großteil der Institute möglich (*n* = 25, 89 %). Durchschnittlich wurden in diesen Instituten ca. 7 Famulantinnen und Famulanten/Jahr betreut. Die Möglichkeit, das Wahltertial im PJ im Fach Rechtsmedizin zu absolvieren, war an 21 Instituten gegeben (75 %). Durchschnittlich nehmen pro Jahr jeweils 3 Studierende dieses Angebot wahr. Ein PJ-Logbuch war an allen 21 Instituten vorhanden.

### Subjektive Beurteilung der Lehre

Die Lehrverantwortlichen wurden gebeten, Aussagen zur eigenen Lehre auf einer 5‑stufigen Likert-Skala einzuordnen, zusätzlich wurde die Option „Kann ich nicht beurteilen“ zur Verfügung gestellt.

**Figure 2:**
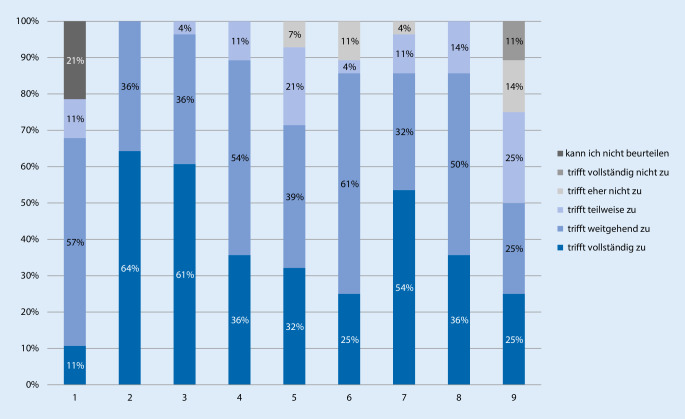

Alle Lehrverantwortlichen stimmten der Aussage vollständig (64 %) oder weitgehend (36 %) zu, dass die angebotenen Lehrveranstaltungen die Studierenden gut auf die jeweiligen fakultätsinternen Prüfungen vorbereiten, und ordneten die Aussage damit einem der beiden positiven Skalenwerte zu (Abb. 2). Demgegenüber sahen nur 11 % (vollständig) bzw. 57 % (weitgehend) der Befragten in den Lehrveranstaltungen eine gute Vorbereitung auf den schriftlichen Teil des 2. Abschnitts der ärztlichen Prüfung und damit die Fragen des Instituts für Medizinische und Pharmazeutische Prüfungsfragen (IMPP), 21 % gaben an, diese Aussage nicht beurteilen zu können.

Insgesamt zeigte sich eine gute Beurteilung der eigenen Lehrveranstaltungen. Der Großteil der Lehrverantwortlichen ordnete Aussagen einem der beiden positiven Skalenpunkte zu (vollständige bzw. weitgehende Zustimmung): 97 % sehen in den Lehrveranstaltungen die wichtigsten rechtsmedizinischen Lernziele widergespiegelt, 90 % bejahen eine gute inhaltliche Abstimmung der einzelnen Lehrveranstaltungen untereinander, 71 % beurteilen die Abstimmung von Unterrichtsinhalten und Lehrformaten als gut, und 86 % sehen die wichtigsten rechtsmedizinischen Lehrinhalte als gut in den Prüfungen abgebildet an. Dementsprechend gaben jeweils 86 % der Befragten an, dass sie in den Lehrveranstaltungen einerseits eine gute Vorbereitung der Studierenden auf die spätere ärztliche Tätigkeit sehen und diese andererseits einen Überblick über die Breite des Faches geben.

Eine kritischere Sicht zeigte sich hinsichtlich der für die Lehre zur Verfügung stehenden Stundenzahl. Diese wurde von nur 50 % der Befragten als ausreichend angesehen, während 25 % die Stundenzahl als nicht ausreichend bewerten.

## Diskussion

Nach den vorliegenden Ergebnissen dominieren bei den fakultätsinternen Prüfungen im Fach Rechtsmedizin faktenbasierte, schriftliche MC-Prüfungen, während praktische Prüfungen deutlich unterrepräsentiert sind. Gegenüber den Ergebnissen aus einer vergleichbaren Befragung aus dem Jahr 2006 [2] zeigte sich hier kaum eine Veränderung. Vor dem Hintergrund, dass in 75 % aller Institute praktischer Unterricht am Leichnam stattfindet [4], weist dieses Ergebnis darauf hin, dass vielerorts ein gewisses „Mismatch“ zwischen Lehr- und Prüfungsmethoden besteht, indem praktische Fertigkeiten durch eine theoretische, faktenbasierte Methode geprüft werden [5]. Dieser Umstand mag sicherlich auch den jeweiligen lokalen Gegebenheiten und Umständen geschuldet sein, da nicht an allen Instituten ausreichende Räumlichkeiten sowie ein Zugang zu prüfungsgeeigneten Verstorbenen vorhanden sind. Es ist jedoch davon auszugehen, dass durch die zu erwartende Novellierung der Approbationsordnung mit Betonung praxisnaher Fertigkeiten und Kenntnisse durch die jeweiligen Fakultäten eine Anpassung verlangt wird, wie dies in anderen klinischen Bereichen sowohl für praktische als auch theoretische Prüfungsformate bereits umgesetzt ist [6, 7]. Es bleibt abzuwarten, ob hier wenigstens teilweise digitale Formate Einsatz finden können [8–10]. Diese verfügen einerseits über den Vorteil eines hohen Maßes an Standardisierung, sie weisen andererseits aber methodisch bedingte Limitationen auf und sind nicht zuletzt an das Vorhandensein technischer Gegebenheiten gebunden. Nach Abschluss der hier vorliegenden Befragung kam es aufgrund der COVID-19-Pandemie vielerorts zu einer Umstellung der Lehr- und Prüfungsformen auf digitale Formate. Es erscheint vorstellbar, dass ein Teil dieser Formate als Supplement Bestand haben oder zu einer bleibenden Veränderung in der Gestaltung von Lehre, Lernen und Prüfen führen wird.

Aufgrund des Überwiegens schriftlicher Prüfungsformate zeigte sich auch eine deutliche Überzahl von Großgruppenprüfungen. Zu begrüßen ist, dass an den meisten Standorten eine computerbasierte Auswertung stattfindet. Dieses Ergebnis spiegelt sich auch in der Angabe wider, dass in 75 % eine Itemanalyse der schriftlichen Prüfungsfragen stattfindet. Die hierbei erhobenen Kennwerte (Schwierigkeit, Trennschärfe) geben den Dozierenden wichtige Hinweise hinsichtlich der Validität und Reliabilität der Prüfungen und können v. a. für eine Verbesserung der Prüfungsfragen, aber auch für die Optimierung der Lehrinhalte genutzt werden.

Sehr erfreulich sind die guten bis sehr guten Evaluationsergebnisse im Fach Rechtsmedizin. Dieses Ergebnis dürfte, neben einem sicherlich zu unterstellenden Interesse der Studierenden an dem Fach und dem Lehrstoff, nicht zuletzt das hohe Engagement der Dozierenden widerspiegeln. Die Lehre spielt in der Rechtsmedizin traditionell eine wichtige Rolle, was sich in zahlreichen Veröffentlichungen zu der Thematik auch in Zeiten widerspiegelt, die teils deutlich vor der in den letzten Jahren an den Fakultäten immer mehr im Zentrum stehenden Medizindidaktik publiziert wurden [11–24]. Für die künftige universitäre Ausrichtung der Rechtsmedizin sollte dieses studentische Interesse weiterhin genutzt werden, ggf. durch die Ausweitung des Lehrangebots auf freiwillige Zusatzangebote [25].

Die Möglichkeit, eine Famulatur im Fach Rechtsmedizin durchzuführen, ist an nahezu 90 % der Institute gegeben; hier zeigte sich eine leichte Erhöhung gegenüber dem Wert aus der Befragung von 2006 [2]. Dieses Ergebnis darf jedoch nicht darüber hinwegtäuschen, dass somit immer noch an ca. 10 % der Institute keine Famulaturen durchgeführt werden können, was am ehesten auf Beschränkungen einer Anerkennung durch die Landesprüfungsämter zurückzuführen sein dürfte.

Demgegenüber zeigte sich, dass die Möglichkeit, an den rechtsmedizinischen Instituten das PJ-Wahlfach durchführen zu können, in den letzten Jahren kontinuierlich zugenommen hat. Während diese Möglichkeit 2006 in nur 35 % der Institute gegeben war [2], stieg der Anteil auf 76 % im Jahr 2016 [26] und bis zum Jahr 2019 auf 89 %, wie die vorliegende Befragung zeigt. Die Ergebnisse zeigen zudem, dass dieses Angebot mit durchschnittlich 3 Studierenden im PJ/Jahr gut genutzt wird und damit den bestehenden Bedarf der Studierenden an dem Angebot widerspiegelt.

Die Selbsteinschätzung der Befragten zu der von ihnen durchgeführten Lehre zeigt auf den ersten Blick ein ähnliches Bild wie die Ergebnisse der Befragung aus dem Jahr 2006 [2]. Eine differenziertere Betrachtung zeigt jedoch einige Unterschiede auf. Vergleichbar zu der damaligen Befragung sind die Ergebnisse zu den Fragen, ob die Lehre eine gute Vorbereitung auf das schriftliche Staatsexamen darstellt (heterogenes Meinungsbild), der eigene Unterricht gut auf die fakultätsinternen Prüfungen vorbereitet und wichtige Lernziele abbildet (hohe Zustimmung), die Prüfung wichtige Lernziele widerspiegelt und die Lehrveranstaltungen insgesamt einen guten Überblick über das Fach bieten (hohe Zustimmung). Zudem bejahte darüber hinaus der Großteil der Befragten, dass die Lehre die Studierenden gut auf ihre spätere ärztliche Tätigkeit vorbereitet. Die Frage, ob die jeweiligen Lehrveranstaltungen gut aufeinander abgestimmt sind, sowie die Frage nach einer Abstimmung von Form und Inhalt der Veranstaltungen wurden jedoch mit einer kritischeren Tendenz beantwortet. Es ist zu vermuten, dass ein gegenüber der damaligen Befragung stärker ausgeprägtes Bewusstsein für medizindidaktische Prinzipien unter den Lehrverantwortlichen hierfür verantwortlich ist, bedingt durch den höheren Anteil an medizindidaktischen Qualifikationen [4]. Ebenso wie für den Bereich Prüfungen ist auch hier davon auszugehen, dass im Rahmen curricularer Reformprozesse, die mit der bevorstehenden Novellierung der Approbationsordnung einhergehen werden, auch die Lehrveranstaltungsformen überdacht und ggf. angepasst werden. Erfreulich ist, dass der Grad der Zufriedenheit mit der für die Lehre zur Verfügung stehenden Stundenzahl zugenommen hat und nur noch in 25 % der Institute der zur Verfügung stehende Zeitrahmen als unzureichend eingeschätzt wird, gegenüber etwa 45 % bei der Befragung 2006 [2].

## Fazit für die Praxis

- Während die Anwendung von Lernzielkatalogen und praxisorientierten Unterrichtsformen in der Rechtsmedizin in den letzten Jahren eine weitere Verbreitung erfahren haben, weisen die Ergebnisse der Befragung auf eine bestehende Inkongruenz im Bereich der Prüfungsformate hin, da hier faktenbasierte Prüfungen noch deutlich überwiegen. Anpassungen durch curriculare Reformprozesse, etwa im Rahmen der anstehenden Novellierung der Approbationsordnung, erscheinen hier erforderlich.
- Die Evaluationsergebnisse der rechtsmedizinischen Lehre spiegeln das studentische Interesse an dem Fach und das hohe Engagement der Lehrenden wider. Letzteres findet auch in der hohen Beteiligung der Institute an dieser und vergangenen Umfragen zum Thema Lehre Ausdruck.
- Die studentische Ausbildung in Famulaturen und praktischem Jahr ist aktuell an etwa 90 % der Institute möglich, und das Angebot wird breit genutzt. Insbesondere hinsichtlich der Möglichkeit, ein PJ-Wahlfach anbieten zu können, hat sich die Situation im Fach Rechtsmedizin in den letzten Jahren deutlich verbessert.
- Die für die Lehre zur Verfügung stehende Stundenzahl wird von einem relevanten Teil der befragten Institute als nichtausreichend bewertet.
